# Opioid Therapy and Implications for Oxidative Balance: A Clinical Study of Total Oxidative Capacity (TOC) and Total Antioxidative Capacity (TAC)

**DOI:** 10.3390/jcm13010082

**Published:** 2023-12-22

**Authors:** Urszula Kosciuczuk, Piotr Jakubow, Katarzyna Tarnowska, Ewa Rynkiewicz-Szczepanska

**Affiliations:** 1 Department of Anaesthesiology and Intensive Therapy, Faculty of Medicine, Medical University of Bialystok, 15-089 Bialystok, Poland; 2 Department of Paediatric Anaesthesiology and Intensive Therapy with Pain Division, Faculty of Medicine, Medical University of Bialystok, 15-089 Bialystok, Poland; piotr.jakubow@umb.edu.pl

**Keywords:** chronic pain, oxidative balance, pharmacotherapy

## Abstract

Background: Opioids are used in pharmacotherapy for chronic pain. The phenomenon of their influence on the oxidative–antioxidant balance is poorly understood. Additionally, little is known about the oxidative status in patients receiving chronic opioid noncancer pain therapy. Methods: The primary goal was to explore oxidative status using the total oxidative capacity (TOC) and total antioxidative capacity (TAC) in patients with chronic lower back pain (LBP) treated with opioids. The secondary task was to present the risk factors connected with the duration of therapy or anthropometric parameters. Plasma TOC and TAC were analyzed in the study group (n = 28), i.e., patients with chronic LBP treated with opioids, and in the control group (n = 11), i.e., healthy volunteers. Results: The TAC was significantly lower in the study group compared to the control group (*p* < 0.05), while the TOC did not differ significantly. A statistically lower TOC for buprenorphine compared to oxycodone (*p* = 0.019) and tramadol (*p* = 0.036) was observed. The TOC did not differ between tramadol and oxycodone. The highest TAC was described for oxycodone, while the TAC for buprenorphine and tramadol was significantly lower in comparison with oxycodone (*p* = 0.007 and *p* = 0.016). The TOC/TAC ratio was higher in patients with nicotinism in both groups.Conclusions: Patients receiving chronic opioid therapy presented a lower antioxidative capacity. There were differences in opioid-induced oxidative imbalance, which is very important clinically. Nicotinism increases the oxidative–antioxidative imbalance. The least oxidative capacity was associated with buprenorphine, while oxycodone showed the greatest antioxidant activity. The most favorable TOC/TAC ratio was observed for buprenorphine. It is suggested that buprenorphine or oxycodone has the best profile, and there is no correlation with the duration of opioid therapy or the opioid dose. However, all opioid substances can potentially enhance the oxidative–antioxidative status.

## 1. Introduction

Chronic lower back pain (LBP) is a common disorder among middle-aged patients and affects up to 23% of the population worldwide, with a peak in the third decade of life. Pharmacotherapy with analgesic ladder principles is a crucial part of multidirectional treatment. Weak or strong opioid drugs are used in cases of moderate or severe pain, respectively [1,2,3]. However, it should be emphasized that the use of opioid drugs requires informed consent and the monitoring of the analgesic and side effects. Long-term opioid pharmacotherapy should be restricted to patients who demonstrate a functional improvement from this type of therapy with a corresponding low risk of abuse [1,2,3,4].

Respiratory depression, mental and cognitive disorders, and constipation are the most common opioid-related side effects. However, the immunosuppressive effect of opioids via the oxidative–antioxidative balance is another important phenomenon. It has been reported that the formation of free radicals with reduced antioxidant activity is increased during opioid administration. This mechanism was described with respect to immunological disturbance, impaired synaptic transmission, pathological microglial activity, and proliferation. The development of opioid tolerance and addiction, opioid hyperalgesia, and cognitive disorders associated with brain plasticity are crucial effects associated with oxidative–antioxidative imbalance [5,6].

The main mechanism of the analgesic effect of opioids is connected to activity at mu receptors (MORs—mu-opioid receptors). MOR activation also results in opioid-induced respiratory depression associated with the inactivation of the pre-Bötzinger complex in the brain stem and neurotransmission disorders, increasing the risk of opioid tolerance. MOR activation has been shown to have the strongest effect on oxidative processes. MOR receptors have a typical structure of G proteins–GPCR–G-protein-coupled receptor, in which activation causes cascading transformations of G protein subunits, resulting in the inhibition of adenylate cyclase (AC) activity, which then causes a decrease in the concentration of cyclic adenosine monophosphate (cAMP), a reduction in protein kinase A activity, and the activation of the mitogen-activated protein kinase (MAPK) cascade. The final effect of multidirectional enzymatic changes in MORs is the transformation of protein transcription with the predominance of oxidative processes, including neuronal nitric oxide synthase (nNOS) and NAD(P)H oxidases and reduced activity of catalase (CAT) and superoxide dismutase (SOD). The effect of opioid-reduced activity of SOD, CAT, and glutathione peroxidase (GPx) has also been described in various tissue and biological materials, including human erythrocytes, plasma, brain tissues, sperm, liver, and kidney. It has been shown that pure MOR agonists have the strongest oxidative effects. It has been observed that agonists of the delta-opioid receptor (DOR) have a neuroprotective effect and lead to an increase in the survivability of neuronal cells. Furthermore, experimental data prove that DOR activation leads to the activation of certain pathways that attenuate oxidative mechanisms and delay the progression of neurodegenerative processes [2,3,4,5,6].

The theory of oxidative stress was introduced in the 1990s and implies multidirectional peroxidative and antioxidant mechanisms, as well as internal and external sources and metabolic consequences. The oxidative condition is dynamic, and it means a balance of oxidative and antioxidative processes. It seems to be neutral in a healthy state. Oxidative reactions produce reactive oxygen species (ROS), reactive nitrogen species, reactive carbonyl species, and metal-free radicals. Antioxidant mechanisms are classified as enzymatic (superoxide dismutase (SOD), catalase (CAT), and glutathione peroxidase (GPx)) or nonenzymatic (vitamin C, vitamin E, glutathione (GSH), thioredoxin system, lipoic acid, carotenoids, and flavonoids), and these control the formation and elimination of free radicals [7,8].

Hyperoxidative activity is a crucial part of the pathophysiological consequences of cardiovascular disorders, metabolic diseases, arthritis, and cancer. It concerns the pathogenesis of acute postoperative pain, cancer pain, chronic noncancer pain, neuropathic and visceral pain, fibromyalgia, and migraines. Hyperoxidative processes following peripheral and basal sensitization have been described as being of great importance. Reactive oxidative markers synergize with other pain neuromediators, including NMDA(N-methyl-D-aspartate), GABA, dopamine, and prostaglandins.

The high reactivity of free radicals and their very short half-life, as well as multidirectional antioxidant enzyme activities, mean that the total description of oxidative stress in scientific studies is complicated. Activated platelets are the main internal source of oxidative and antioxidative mediators, and serum is the less proper biological fluid used. Therefore, measurements of the plasma’s total oxidative capacity (TOC) and total antioxidant capacity (TAC) are preferred for a better understanding of the mechanisms of oxidative–antioxidative balance and provide new clinically important information [1,2,3,4,5].

The phenomenon of opioid-induced oxidative–antioxidative imbalance is poorly understood. Some studies have shown marked differences in parameters of oxidative stress between opioid abuse and healthy volunteers. However, little is known about the oxidative status in patients with chronic noncancer pain treated with opioids. It is not known whether doses of different opioids improve or exacerbate the oxidative imbalance.

The primary aim of the present study was to investigate levels of oxidative stress markers in patients with LBP with monotherapy using opioids. Based on previous related studies, our a priori hypothesis was that the specific oxidative stress markers TAC and TOC would be elevated compared to controls. A secondary aim was to determine whether markers of oxidative stress are associated with the duration of therapy. Although few studies have investigated markers of oxidative stress in relation to different opioid substances in abuse and their results have been inconsistent, our exploratory hypothesis was that oxidative stress in opioid therapy forchronic pain would depend on the type of substance.

## 2. Materials and Methods

### 2.1. Patient Enrolment and Clinical Specimens

This study was approved by the Ethical Committee for Human Studies of the Medical University of Bialystok, Poland, and was registered on ClinicalTrials.gov (NCT04227223). The inclusion criteria were as follows: age 18–80 years old with LBP, with opioid therapy resulting in a stable NRS (numerical rating scale) value less than 4. Patients more than 80 years old with cognitive disorders who were receiving anti-inflammatory therapy (nonsteroidal anti-inflammatory drugs (NSAIDs), steroids, and other immune-modulating agents) were excluded. The control group included healthy volunteers. Pain related to spinal tissues, as well as radiculopathy pain, with a duration over 3 months was adopted as the definition of chronic LBP.

Firstly, medical histories in the pain clinic were analyzed, and the number of patients with chronic LBP pain with opioid therapy was recorded (n = 77). There were 35 patients with chronic LBP with opioid monotherapy and 42 patients with polytherapy. All patients provided informed consent and were recruited through direct contact with medical staff. The study involved 50 patients, 35 in the study group and 15 in the control group.

### 2.2. Blood Sampling and Oxidative STRESS Measurements

All blood samples were processed strictly following the same protocol. The patients continued their treatment with opioids and took the prescribed doses on the day the biological material was collected. In the morning, after 6 h of fasting and abstinence from smoking, blood samples were aseptically drawn into tubes containing ethylenediaminetetraacetic acid (2.7 mL EDTA vacutainers) via cubital vein puncture (gauge of needle 21G, BD Vacutainer, Becton Dikinson, Franklin Lakes, NJ, USA). The plasma was isolated after centrifugation (1000× *g* at 2–8 °C within 30 min of collection) and immediately stored at −80 °C in aliquots of 300 microliters in Eppendorf tubes until required. The determinations were performed after 2 weeks in the Department of Biochemical Diagnostics. The plasma TOC and TAC levels were analyzed using photometric methods (PerOx, ImAnOx, Immundiagnostik AG, Bensheim, Germany) [9]. 

The determination of the total antioxidative capacity (TAC, ImAnOx) was performed using the reaction of antioxidants in the sample with a defined amount of exogenously provided hydrogen peroxide (H_2_O_2_). The antioxidants in the sample eliminated a certain amount of the hydrogen peroxide provided. The residual H_2_O_2_was determined photometrically using an enzymatic reaction that involved the conversion to a colored product. After the addition of a stop solution, the samples were measured at 450 nm in a microtiter plate reader. The quantification was performed using a delivered calibrator. The limit of the TAC detection was 130 µmol/L. The difference between the applied and measured peroxide concentration in a defined time period is proportional to the reactivity of the antioxidants of the sample (antioxidative capacity). The difference in the sample values with and without the enzyme is inversely proportional to the antioxidative capacity. To obtain the ∆OD, one subtracts the OD values of the samples without the enzymes from the OD values of the samples with the enzyme. The antioxidative capacity was calculated according to the following formula:

antioxidative capacity [µmol/L] = 392-(392-calibrator concentration) × [∆ODsample/∆OD calibrator]

Based on Immundiagnostik studies of the EDTA plasma and serum of healthy persons, the following reference values were estimated: low antioxidative capacity, <280 µmol/L; middle antioxidative capacity, 280–320 µmol/L; high antioxidative capacity, >320 µmol/L; with a mean value of 305 µmol/L.

The PerOx test (TOC) measures the activity of lipid peroxides. The determination of the peroxides was performed using the reaction of a peroxidase with peroxides in the sample followed by the conversion to a colored product. Measurement 1 presents the initial absorption of the samples in the ELISA reader at 450 nm. Measurement 2 was performed immediately after the addition of the stop solution at 450 nm in the ELISA reader. The difference between measurements 1 and 2 was directly and linearly proportional to the peroxide content of the sample. The reference ranges were as follows: EDTA-plasma < 200 µmol/L, low oxidative stress; 200–350 µmol/L, moderate oxidative stress; >350 µmol/L, high oxidative stress; linearity up to 800 µmol/L and a detection limit of 7 µmol/L [9].

### 2.3. Statistical Analysis

A statistical analysis was performed using Statistica 13.1 (StatSoft, Krakow, Poland). The Kolmogorov–Smirnov test showed no normal distribution of the obtained results; therefore, nonparametric methods were implemented. The data were expressed as the median, minimum, and maximum values and interquartile ranges. The Mann–Whitney U test was applied to analyze quantitative values between the study and control groups. The associations between the measured parameters were tested using the Spearman rank correlation coefficient. Statistical significance was established at *p* ≤ 0.05. This was a pilot study; therefore, the sample size was determined as 35 subjects in the experimental group.

## 3. Results

### 3.1. Characteristics of the Groups

**Figure 1:**
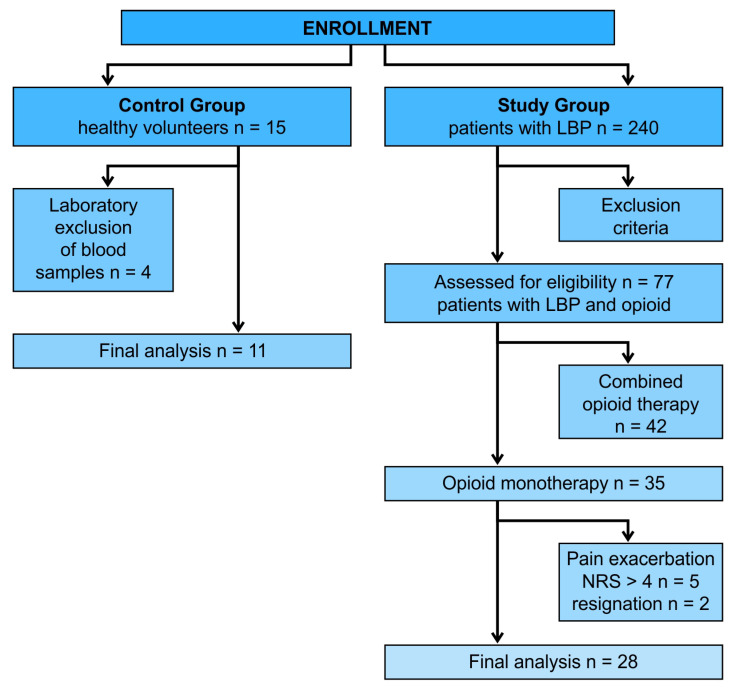
Flowchart–initial cohort in study and control groups.

Data from 39 patients were used for analysis, including 28 from the study group (10 men and 18 women with a median age of 70.5 years and a median BMI of 27.2) and 11 from the control group (six men and five women with a median age of 64.3 years and a median BMI of 26.7). The recruitment process is presented in Figure 1. Anthropometric data did not differ between groups (Table 1).

In the study group, five patients were excluded from the study due to the exacerbation of clinical pain NRS > 4 and taking nonsteroidal anti-inflammatory drugs, and two patients resigned. In the control group, data from four patients were excluded due to insufficient vein punction and collected blood volume to perform the analyses. The median duration of opioid therapy in the study group was 26 months (min–max range of 12–34), with a morphine equivalent of 40 mg (min–max range of 7.5–160).

### 3.2. TAC and TOC Concentrations

**Figure 2:**
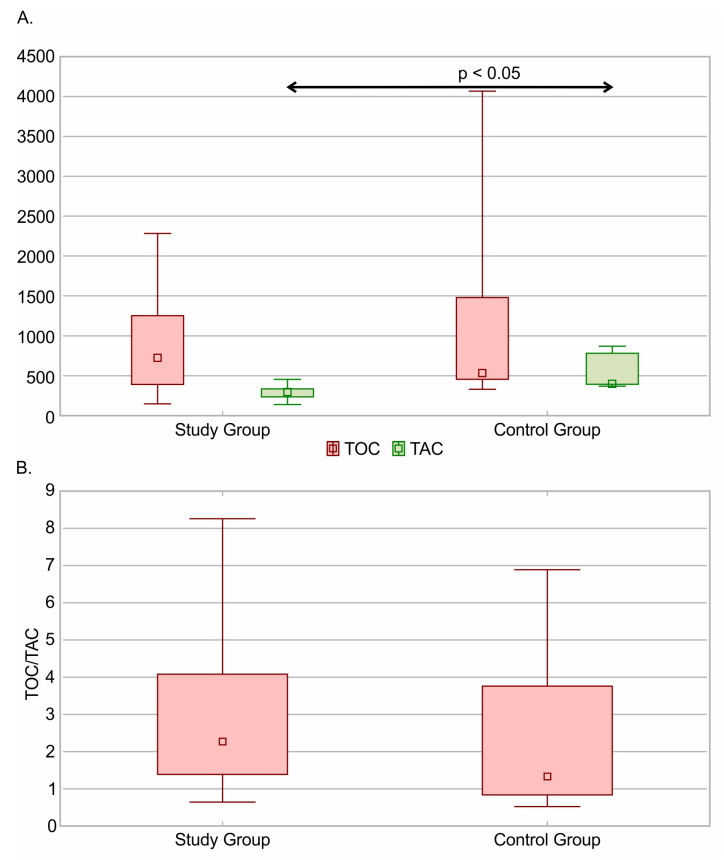
(**A**) Total Oxidant Capacity [TOC] and Total Antioxidant Capacity [TAC] and (**B**) TOC/TAC ratio in Study and Control Group.The median, minimum and maximum, and interquartile ranges are presented.

The TAC was significantly lower in patients undergoing opioid pain therapy (median 295.15 µmol/L (IQR: 235.25–335.85 µmol/L)) than in the control group (median 399.33 µmol/L (IQR: 393.56–782.64 µmol/L)) (*p* < 0.001). The median TOC value in the study group was 723 µmol/L (IQR: 391.64–1253.84 µmol/L) and did not differ significantly from that in the control group (median 533 µmol/L (IQR: 455.79–1480.31 µmol/L)). The TAC and TOC values and the TOC/TAC ratio are presented in Figure 2.

**Figure 3:**
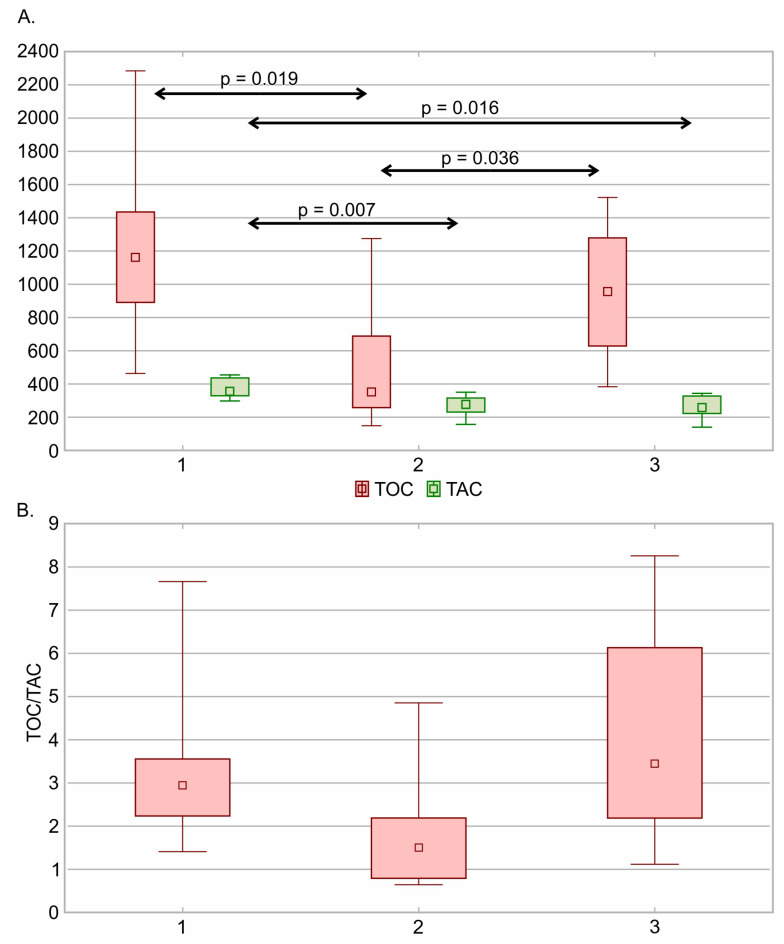
(**A**) Total Oxidant Capacity [TOC] and Total Antioxidant Capacity [TAC] and (**B**) TOC/TAC ratio in different opioid substances. 1—oxycodone, 2—buprenorphine, 3—tramadol. The median, minimum and maximum, and interquartile ranges are presented.

The study presented a significantly lower TOC value in the group using buprenorphine therapy (n = 10) than in the group using oxycodone (n = 6) (*p* = 0.019) and tramadol (n = 8) (*p* = 0.036). Indeed, the TOC did not differ between tramadol (median 956.00 µmol/L (IQR: 628.79–1278.97 µmol/L)) and oxycodone (median 352.10 µmol/L (IQR: 257.64–688.41 µmol/L)). Furthermore, we observed the highest value of TAC in the group of patients undergoing oxycodone therapy (median 354.93 µmol/L (IQR: 329.13–436.81 µmol/L)). The analysis of the TAC in different opioid substances showed that median TAC values in the buprenorphine (median 277.38 µmol/L (IQR: 231.05–315.61 µmol/L)) and tramadol (median 257.94 µmol/L (IQR: 222.13–327.68 µmol/L)) groups were significantly lower than in the oxycodone group (*p* = 0.007 and *p* = 0.016, respectively). In the detailed analysis, we did not include patients using morphine (n = 3) and fentanyl (n = 1) due to the small sample size. The TAC and TOC values and the TOC/TAC ratio in the oxycodone, buprenorphine, and tramadol groups are presented in Figure 3.

The analysis did not present any correlation between TAC and TOC with the duration of opioid therapy and the opioid dose expressed as morphine equivalents. In the study group with concomitant nicotinism, the individual pharmacological substances were as follows: buprenorphine (five patients), tramadol (three patients), oxycodone (five patients), and morphine (three patients). The group without nicotinism included buprenorphine (five patients), tramadol (five patients), oxycodone (one patient), and fentanyl (one patient).

**Figure 4:**
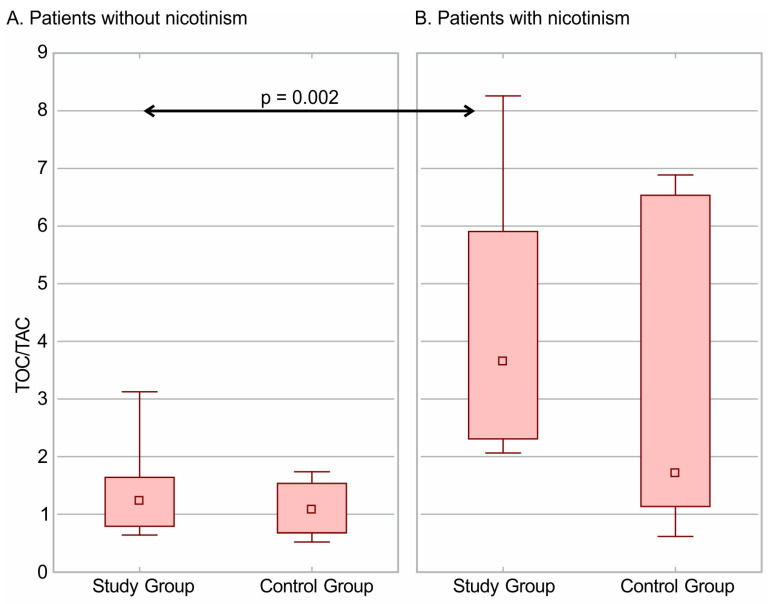
The values TOC/TAC ratio in patients without nicotinism (**A**) and with nicotinism (**B**) in Study and Control Group. The median, minimum and maximum, and interquartile ranges are presented.

The median values of the TOC/TAC ratio were higher in patients with nicotinism in both groups. Moreover, significant differences were noticed in the TOC/TAC ratio in the study group. The values of the TOC/TAC ratio in patients without nicotinism and with nicotinism in the study and control groups are presented in Figure 4.

## 4. Discussion

Epidemiological observations indicate that opioid drug administration has been increasing in recent years. This trend has occurred in central and western Europe, Scandinavia, and the countries of America [10]. Epidemiologic analyses have shown that up to 30% of patients with LBP are prescribed opioids in family practice, and up to 60% receive such prescriptions in emergency departments [11]. Many authors have presented only the short-term efficacy of opioid therapy in pain relief with functional improvement. Nevertheless, the long-term efficacy and safety are not well known. Indeed, an increased risk of abuse and tolerance is considered [1,2,3,4]. One-third of patients have been described as benefitting from long-term opioid therapy, and it has been suggested that this type of therapy be considered in some carefully selected and monitored patients [12]. Opioids provide clinically relevant pain relief in 30% of patients with a reduction in disability in chronic lower back pain [13,14,15,16]. The most important recommendation is not to use opioids as a first-line therapy [3]. In our clinical observations, 30% of patients received opioid-based therapy in the pain clinic, while 20% started opioid therapy before consultation in the pain clinic, and 70% of patients were treated with opioid therapy with coanalgesics.

Different oxidative stress markers connected to opioid substances were detected. The total oxidative capacity (TOC) and total antioxidative capacity (TAC) present new possibilities for a fully oxidative–antioxidative state. In our study, we used an original TOC/TAC ratio to better understand the dependence of these two opposite situations. Leventelis et al. showed that serum GSH concentration and serum CAT activity were significantly reduced in opium-dependent patients. The authors concluded that buprenorphine has greater antioxidant activity than other opioid substances. The study also compared oxidative stress with respect to carbonyl and thiobarbituric acid reactive substances, which were significantly higher in the study group, but there were no differences between buprenorphine and methadone. Moreover, no significant differences in total antioxidant capacity between groups were noted [17]. Salarian et al. observed that serum SOD and CAT activities in opium abuse patients were reduced, and methadone therapy resulted in the normalization of parameters after the 14th day of therapy. However, similar changes were not observed in other components of oxidative stress, such as glutathione and malondialdehyde (MDA) [18].

In our study, the secondary point was to analyze anthropometrical data or details of opioid therapy with respect to the presence of risk factors. Nicotinism is a well-known negative risk factor in oxidative stress profiles. It was noted that the serum TAC value (median 589 µmol/L) was significantly lower and the TOC value (median 808 µmol/L) was significantly higher in patients with nicotinism than in healthy volunteers at 675 and 596 µmol/L, respectively [8]. In the group of patients with nicotinism, a significantly reduced CAT activity was also noted (274 vs. 216 k/mL), and in the group of opioid users, the SOD activity was significantly lower than in healthy controls (25 vs. 12 U/mg) [18]. In our study, the median TOC value was 1220 µmol/L, while the median TAC value was 287 µmol/L in patients with nicotinism in the study group. Moreover, nicotinism exacerbates oxidative disturbances during opioid therapy. These results are crucial in clinical practice.

Sadat-Shirazi et al. described significantly reduced SOD activity and increased MDA concentration in the prefrontal brain region in opium-addicted patients [19]. You Cai et al. described oxidative stress in hippocampal neurons in an animal model with morphine therapy, leading to a decrease in the density of excitatory synapses while increasing inhibitory synapses [20]. The opioid stimulation of ROS production has been described in animal models, as well as in different cell lines. This phenomenon was observed in microglial cells and macrophages [21]. Additionally, many experimental animal studies have shown that morphine increases markers of oxidative stress in the cerebral cortex and serum [22]. Moreover, significantly increased serum MDA and decreased SOD and CAT activity were observed during tramadol treatment [23]. Indeed, a significant increase in carbonylated proteins in the rat brain cortex and serum after 30 days of oxycodone treatment was also reported [24].

The cellular mechanisms of opioid-induced immunomodulation and oxidative status do not follow a constant pattern. Receptor-internalizing agonists (fentanyl and sufentanyl) present high potency to activate oxidative processes via phospholipase D2 (PLD2) and NADH/NADPH-mediated ROS synthesis. MOR activation has been shown to have the strongest effect on oxidative processes. Agonists of the delta-opioid receptor (DOR) have a neuroprotective effect and lead to an increase in neuronal cell survivability. Further, DOR activation leads to the attenuation of oxidative mechanisms and delays the progression of neurodegenerative processes [25,26,27,28,29,30,31]. Our study was conducted on opioids without internal activity (morphine, buprenorphine, tramadol, and oxycodone), andwe described differences in the oxidative/antioxidative balance for opioid substances with the best TOC/TAC ratio for buprenorphine therapy.

Oxidative stress depends on many personal (age and BMI), environmental (social and emotional conditions), and medical factors (duration of opioid therapy, total dose of opioids, and renal and liver function) [32,33,34,35,36,37,38,39]. Our study noticed important results in nicotinism and indicated no correlation between oxidative stress, the duration of opioid therapy, and the dose of opioids.

Tramadol[(±)cis-2-[(dimethylamino)methyl]-1-(3-methoxyphenyl)-cyclohexanol hydrochloride)] has a multidirectional mechanism of action, and it is a popular analgesic with a low risk of opioid side effects. Tramadol has a unique analgesic action as a central opioid agonist and central reuptake inhibitor of norepinephrine and serotonin. Enantiomer (+)tramadol is a selective mu-receptor agonist with a mu activity approximately10-fold less than that of codeine, and it inhibits serotonin reuptake. Enantiomer (-)tramadol inhibits norepinephrine reuptake. The additional ability of selective serotonin reuptake inhibitors(SSRIs) to selectively regulate serotonin concentration is very important. Many authors have reported that SSRI strongly supports the antioxidant effect, and the SSRI mechanism is used as a coanalgesic [40]. In our study, tramadol showed the lowest antioxidant and intermediate oxidative potency. Perhaps this indirect place between buprenorphine and oxycodone was related to an additional mechanism of serotonin regulation. In the final evaluation, tramadol had the highest TOC/TAC ratio [40,41].

This study had several limitations that should be considered. Firstly, the small sample size and associated low statistical power may have affected the results of this study. Secondly, the analysis was limited to selected opioid substances. However, it should be emphasized that the use of opioid drugs as an element of chronic pain therapy requires an analysis of their benefits and risks. Moreover, it is not a first-line therapy, and the group of patients with opioid monotherapy is very limited, and recruitment is difficult. Polytherapy is a more frequent method to reduce opioid tolerance. In our study, 64% of patients received polytherapy with coanalgesics to reduce the doses of opioids and inhibit the development of tolerance. In the detailed analysis, we did not include patients using morphine (n = 3) and fentanyl (n = 1) due to the small sample size.

Due to the limitations of this study, it is necessary to confirm the results in a larger cohort. This study was probably underpowered to answer the research question. As an extension of this study, we see a need to examine other effects of the long-term use of opioids, including their impact on mental health, including the risk of developing depression and anxiety, as well as memory and concentration processes (for example, the Wisconsin Card Sorting Test, PSS, and the GAD-7 score).

## 5. Conclusions

In conclusion, the present study suggests that patients with long-term opioid therapy showed severe oxidative–antioxidative impairments compared to healthy participants. Also, our study presented differences in opioid-induced oxidative imbalance. Notably, the least oxidative disturbances were associated with buprenorphine, while oxycodone had the greatest antioxidant activity. Finally, the most favorable TOC/TAC ratio was observed for buprenorphine. In addition, nicotinism increases oxidative stress in patients taking opioids for the treatment of chronic pain. It is suggested that buprenorphine and oxycodone have the best profile, and it has no correlation with the duration of opioid therapy or the opioid dose. However, all opioid substances can potentially enhance the oxidative–antioxidative status.

## Figures and Tables

**Table 1:** Demographic characteristics in study and control group.

	Study Group	Control Group	*p*–Value
**n**	28	11	*p* < 0.05
**men/women**	10/18	6/5	*p* < 0.05
**age (years)**	70.5	64.3	*p* < 0.05
**BMI**	27.2	26.7	*p* < 0.05

## Data Availability

Data are available from authors upon special request.

## References

[1] Silva C., Oliviera D., Pestana-Santos M., Portugal F., Capelo P. (2022). Chronic non-cancer pain in adolescens: A narrative review. Braz. J. Anaesth..

[2] Esch T., Kream R., Stefano G.B. (2020). Emerging regulatory roles of opioid peptides, endogenous morphine, and opioid receptor subtypes in immunomodulatory processes: Metabolic, behavioral, and evolutionary perspectives. Immunol. Lett..

[3] Santoni A., Mercadante S., Arcuri E. (2021). Chronic cancer and non-cancer pain and opioid-induced hyperalgesia share common mechanisms: Neuroinflammation and central sensitization. Minerva Anestesiol..

[4] Cavallone L.F., Montana M.C. (2021). Pain, the immune system and opioids are cross-talking: Are we just listening in, or can we shape the conversation?. Minerva Anesthesiol..

[5] Shakouri S.K., Dolatkhah N., Omidbakhsh S., Pishgahi A., Hashemian M. (2020). Serum inflammatory and oxidative stress biomarkers levels are associated with pain intensity, pressure pain threshold and quality of life in myofascial pain syndrome. BMC Res. Notes.

[6] Vaegter H.B., Ussing K., Johansen J.V., Stegemejer I., Palsson T.S., O’Sullivan P., Kent P. (2019). Improvements in clinical pain and experimental pain sensitivity after cognitive functional therapy in patients with severe persistent low back pain. Pain Rep..

[7] Danielson E.C., Mazurenko O., Andraka-Christou B.T., DiIulio J., Downs S.M., Hurley R.W., Harle C.A. (2019). An Analysis of Primary Care Clinician Communication About Risk, Benefits, and Goals Related to Chronic Opioid Therapy. MDM Policy Pract..

[8] Buico A., Cassino C., Ravera M., Betta P.-G., Osella D. (2009). Oxidative stress and total antioxidant capacity in human plasma. Redox Rep..

[9] http://www.immundiagnostik.com/en/home.

[10] Denawa Y., Kurtz W., Conermann T. (2019). The Social and Functional Implications of High- Versus Low-Dose Opioids on Chronic Non-Cancer Pain. Pain Phycisian.

[11] Kamper S.J., Logan G., Copsey B., Thompson J., Machado G.C., Abdel-Shaheed C., Williams C.M., Maher C.G., Hall A.M. (2019). What is usual care for low back pain? A systematic review of health care provided to patients with low back pain in family practice and emergency departments. Pain.

[12] Bialas P., Maier C., Klose P., Häuser W. (2020). Efficacy and harms of long-term opioid therapy in chronic non-cancer pain: Systematic review and meta-analysis of open-label extension trials with a study duration ≥26 weeks. Eur. J. Pain.

[13] Petyke F., Klose P., Welsch P., Sommer C., Häuser W. (2020). Opioids for chronic low back pain: An updated systematic review and meta-analysis of efficacy, tolerability and safety in randomized placebo-controlled studies of at least 4 weeks of double-blind duration. Eur. J. Pain.

[14] Oliveira C.B., Maher C.G., Pinto R.Z., Traeger A.C., Lin C.W.C., Chenot J.F., van Tulder M., Koes B.W. (2018). Clinical practice guidelines for the management of non-specific low back pain in primary care: An updated overview. Eur. Spine J..

[15] Skrabalova J., Drastichova Z., Novotny J. (2013). Morphine as a potential oxidative stress-causing agent. Mini-Rev. Org. Chem..

[16] Tsai M.C., Huang T.L. (2017). Brain-derived neutrophic factor (BDNF) and oxidative stress in heroin-dependent male patients undergoing methadone maintenance treatment. Psychiatry Res..

[17] Leventelis C., Goutzourelas N., Kortsinidou A., Spanidis Y., Toulia G., Kampitsi A., Tsitsimpikou C., Stagos D., Veskoukis A.S., Koureta D. (2019). Buprenorphine and methadone as opioid maintenance treatments for heroin-addicted patients induce oxidative stress in blood. Oxidative Med. Cell. Longev..

[18] Salarian A., Kadkhodaee M., Zahmatkesh M., Seifi B., Bakhshi E., Akhondzadeh S., Adeli S., Askari H., Arbabi M. (2018). Opioid use disorder induces oxidative stress and inflammation. The attenuation effect of methadone maintenance treatment. Iran. J. Psychiatry.

[19] Sadat-Shirayi M.S., Yarrisdast M.R., Ashabi G. (2020). Oxidative stress enzymes are changed in opioid abusers and multidrug abusers. J. Clin. Neurosci..

[20] Cai Y., Yang L., Hu G., Chen X., Niu F., Yuan L., Liu H., Xiong H., Arikkath J., Buch S. (2016). Regulation of morphine-induced synaptic alternations—Role of oxidative stress, ER stress and autophagy. J. Cell Biol..

[21] Bhat R.S., Bhaskaran A., Mongia N. (2004). Morphine-induced macrophage apoptosis: Oxidative stress and strategies for modulation. J. Leukoc. Biol..

[22] Karkhah A., Ataee R., Ataie A. (2017). Morphine pre-and post-conditioning exacerbates apoptosis in rat hippocampus cells in a model of homocysteine-induced oxidative stress. Biomed. Rep..

[23] Awadalla E.A., Salah-Eldin A.E. (2016). Molecular and histological changes in cerebral cortex and lung tissues under the effect of tramadol treatment. Biomed. Pharmacother..

[24] Fan R., Schrott L.M., Snelling S., Felty J., Graham D., McGauly P.L., Arnold T., Korneeva N.L. (2020). Carbonyl-protein content increases in brain and blood of female rats after chronic oxycodone treatment. BMC Neurosci..

[25] Koch T., Seifert A., Wu D.-F., Rankovic M., Kraus J., Börner C., Brandenburg L.-O., Schröder H., Höllt V. (2009). μ-opioid receptor-stimulated synthesis of reactive oxygen species is mediated via phospholipase D2. J. Neurochem..

[26] Sacredote P. (2008). Opioid-induced immunosuppression. Curr. Opin. Support. Palliat. Care.

[27] Kosciuczuk U., Knapp P., Lotowska-Cwiklewska A.M. (2020). Opioid-induced immunosuppression and carcinogenesis promotion theories create the newest trend in acute and chronic pain therapy. Clinics.

[28] Plein L.M., Reither H.L. (2018). Opioids and the immune system—Friend or foe. Br. J. Pharmac..

[29] Arezoomandan M., Zhiani R., Mehrzad J., Motavalizadehkakhy A., Eshrati S., Arezoomandan R. (2022). Inflammatory, oxidative stress and cognitive functions in patients under maintenance treatment with methadone or buprenorphine and healthy subjects. J. Clin. Neurosci..

[30] Tobore T.O. (2021). Towards a comprehensive theory of non-cancer acute and chronic pain management:the critical role of reactive oxygen and nitrogen species in pain, and opioid dependence, addiction, hyperslgesia, and tolerance. Adv. Redox Res..

[31] Quilan J., Levy N., Lobo D.N., Macintyre P.E. (2021). Preoperative opioid use: A modifiable risk factor for poor postoperative outcomes. Br. J. Anaesth..

[32] Snidvongs S., Mehta V. (2012). Recent advances in opioid prescription for chronic non-cancer pain. Postgrad. Med. J..

[33] Newman M., Connery H., Boyd J. (2022). Opioids and Vitamin C: Known interactions and potential for redox-signaling crosstalk. Antioxidants.

[34] Malagfolia V., Ilari S., Vitiello L., Tenti M., Balzani E., Muscoli C., Raffaeli W., Bonci A. (2022). The interplay between cgronic pain, opioids, and the immune system. Neuroscientist.

[35] Foster N.E., Anema J.R., Cherkin D., Chou R., Cohen S.P., Gross D.P., Ferreira P.H., Fritz J.M., Koes B.W., Peul W. (2018). Prevention and treatment of low back pain: Evidence, challenges, and promising directions. Lancet.

[36] Will J.S., Bury D.C., Miller J.A. (2018). Mechanical Low Back Pain. Am. Fam. Physician.

[37] Golob A.L., Wipf J.E. (2014). Low Back Pain. Med. Clin. N. Am..

[38] Maher C., Underwood M., Buchbinder R. (2017). Non-specific low back pain. Lancet.

[39] Larjow E., Papavasiliou E., Payne S., Scholten W., Radbruch L. (2016). A systematic content analysis of policy barriers impeding access to opioid medication in Central and Eastern Europe: Results of ATOME. J. Pain Symptom Manag..

[40] Gałecki P., Mossakowska-Wójcik J., Talarowska M. (2018). The anti-inflammatory mechanisms od antidepressants—SSRIs and SNRIs. Prog. Neuro Psychopharmacol. Biol. Psychiatry.

[41] Beakley B.D., Kaye A.M., Kaye A.D. (2015). Tramadol, pharmacology, side-effects and serotonin syndrome: A review. Pain Physician.

